# Supplementary material for: Fluorescence Enhancement by Calixarene Supramolecular Aggregate
Source: Molecules. 2020 Dec 14;25(24):5912. doi: 10.3390/molecules25245912 (PMC7764848; doi:10.3390/molecules25245912)
Supplement: Supplementary file 1 [file molecules-25-05912-s001.pdf]

Supporting information

Fluorescence Enhancement by Calixarene Supramolecular Aggregate

Xin-Yue Hu, Yu-Ying Wang, Hua-Bin Li * and Dong-Sheng Guo *

Key Laboratory of Functional Polymer Materials (Ministry of Education), State Key Laboratory of Elemento-Organic Chemistry, College of Chemistry, Nankai University, Tianjin 300071, China; huxinyue@nankai.edu.cn (X.-Y.H.); kindwyy@mail.nankai.edu.cn (Y.-Y.W.)

* Correspondence: lihuabin@nankai.edu.cn (H.-B.L.); dshguo@nankai.edu.cn (D.-S.G.)

Table contents

1. Synthesis and ^{1}H-NMR spectra calixarenes 2

2. ITC spectra 6

3. Fluorescence quantum yield and fluorescence spectra. 9

References 10

1. Synthesis and ^{1}H-NMR spectra calixarenes

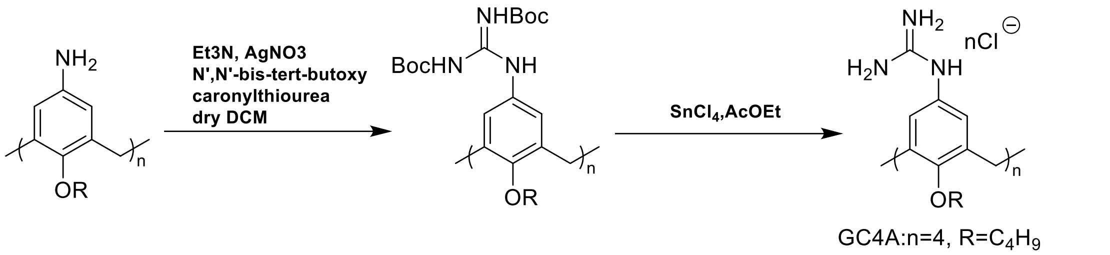

General Procedure for synthesis the Boc Protected guanidinium calixarenes: to the solution of amino calixarene[1] in dry DCM, N′, N′-bis-tert-butoxycarbonylthiourea (1.5 eq per amino group), $AgNO_3$ (1.5 eq per amino group) and Et_3N were added and the mixture was stirred for 48 h at room temperature. The solvent was removed in vacuo and the residue was purified by column chromatography to obtain a white powder.

General Procedure for removal the Boc Protecting group: $SnCl_4$ was added to a solution of the protected guanidinium calixarenes in AcOEt. The mixture was stirred for 3 h at room temperature and the solvent was removed in vacuo. The residue was dissolved in CH_3OH, then large amounts of diethyl ether was added to obtain white powder GC*n*A.

GC5A:^{1}H-NMR (400 MHz, DMSO, σ): 9.62 (s, 5H, NH), 7.35 (s, 20H, NH), 6.95 (s, 10H, ArH), 4.46 (d, *J* = 13.61 Hz, 5H,Ar-CH_2-Ar), 3.80 (t, *J* = 7.74 Hz, 10H, CH_2-O-Ar), 3.46 (d, *J* = 13.58 Hz, 5H; ArCH_2-Ar), 1.91 (p, *J* = 7.54 Hz, 10H), 1.61 (m, 5H), 1.32 (m, 10H), 0.94 (d, *J* = 6.61 Hz, 30H).

GC4A:^{1}H-NMR (400 MHz, DMSO, σ): 9.59 (s, 4H, NH), 7.22 (s, 16H, NH), 6.56 (s, 8H, ArH), 4.33 (d, *J* = 13.1 Hz, 4H, Ar-CH_2-Ar), 3.89 (t, *J* = 7.2 Hz, 8H, CH_2-O-Ar), 3.49 (d, *J* = 14.2 Hz, 4H, Ar-CH_2-Ar),1.87 (dt, *J* = 14.2, 7.2 Hz, 8H), 1.45 (m, 8H), 1.07 (4H), 0.98 (t, *J* = 7.4 Hz,12H).

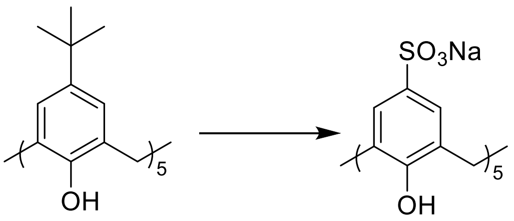

p-tertbutylcalix[5]arene[2] (1.0 g, 1.23 mol) was suspended with H_2SO_4 (98%, 10 mL) at 80–90 °C for 4 h, with periodic checking of the reaction mixture forsolubility in water, resulted in a dark solution in which most of thecalixarene had dissolved. The mixture was quenched in ice (10 g) and filtered. Addition of NaCl (0.25 g) to the filtrate resulted in the slow deposition of the product as colorless prisms which were collected by filtration and dried in vacuo at 100 °C for 72 h.

SC5A:^{1}H-NMR (400 MHz, D_2O, σ): 7.57 (s, 10H), 3.89 (s, 10H)

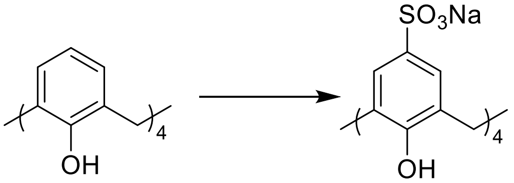

0.5 g of calix[4]arene[3] was suspended in 5 mL H_2SO_4 (98%) at room temperature and stirred for 1 h. The reaction was an intensely exothermic reaction. After being cooled to room temperature, 10 mL saturated NaCl solution was added dropwise into the solution. The solution was boiled and refluxed for 5 minutes then cooled to room temperature. A water-soluble precipitate was obtained by filtration and the crude solid was purified by recrystallization.

SC4A:^{1}H-NMR (400 MHz, D_2O, σ): 7.53 (s, 8H), 3.97 (s, 8H).

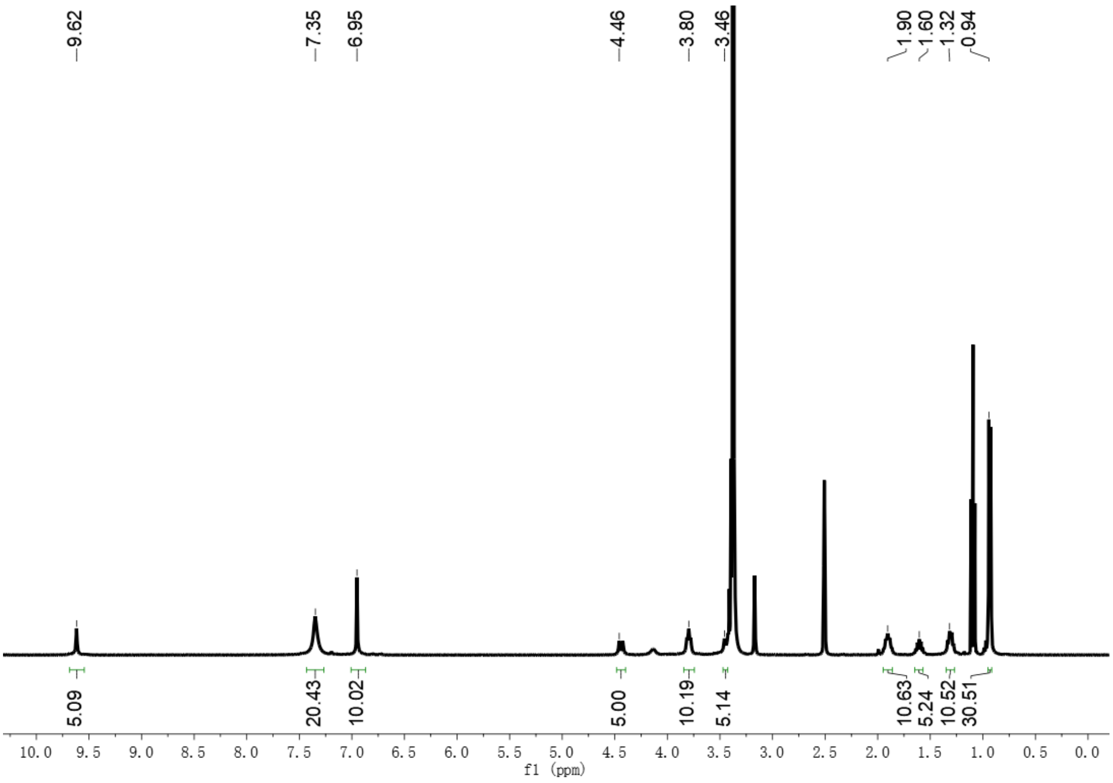

Figure S1. ^{1}H-NMR spectrum of GC5A in DMSO-d_6 (400 MHz, 298.15K).

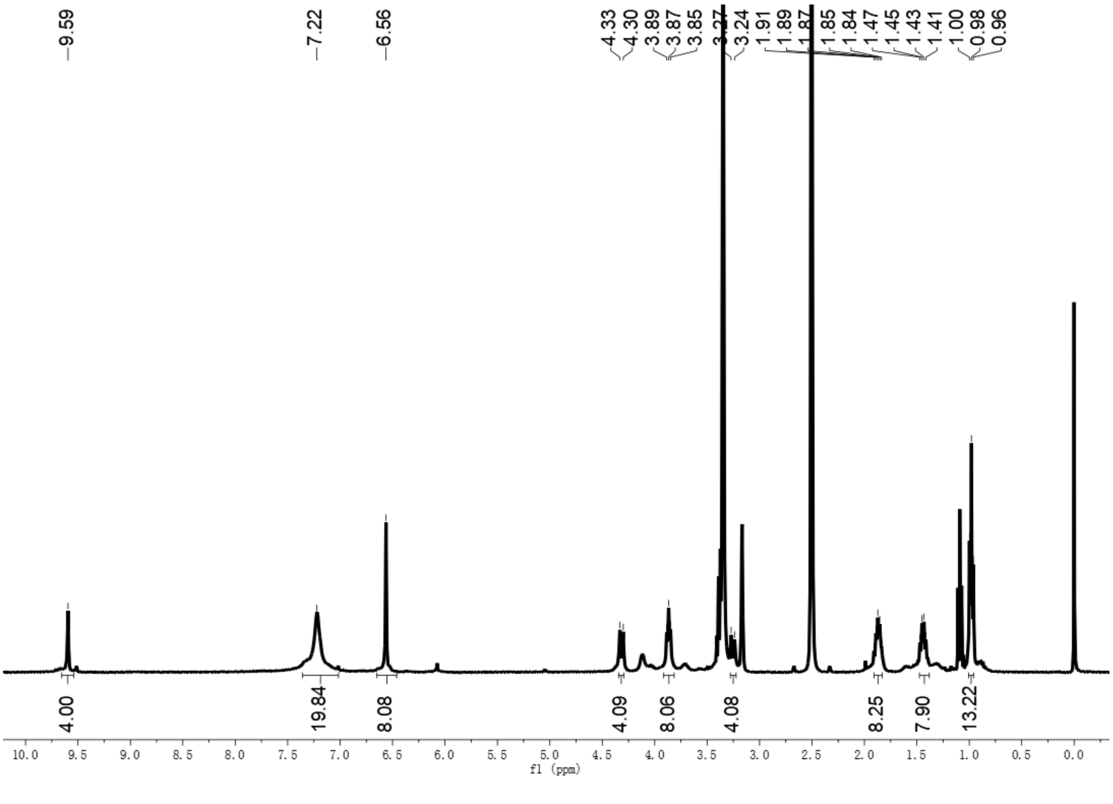

Figure S2. ^{1}H-NMR spectrum of GC4A in DMSO-d_6 (400 MHz, 298.15K).

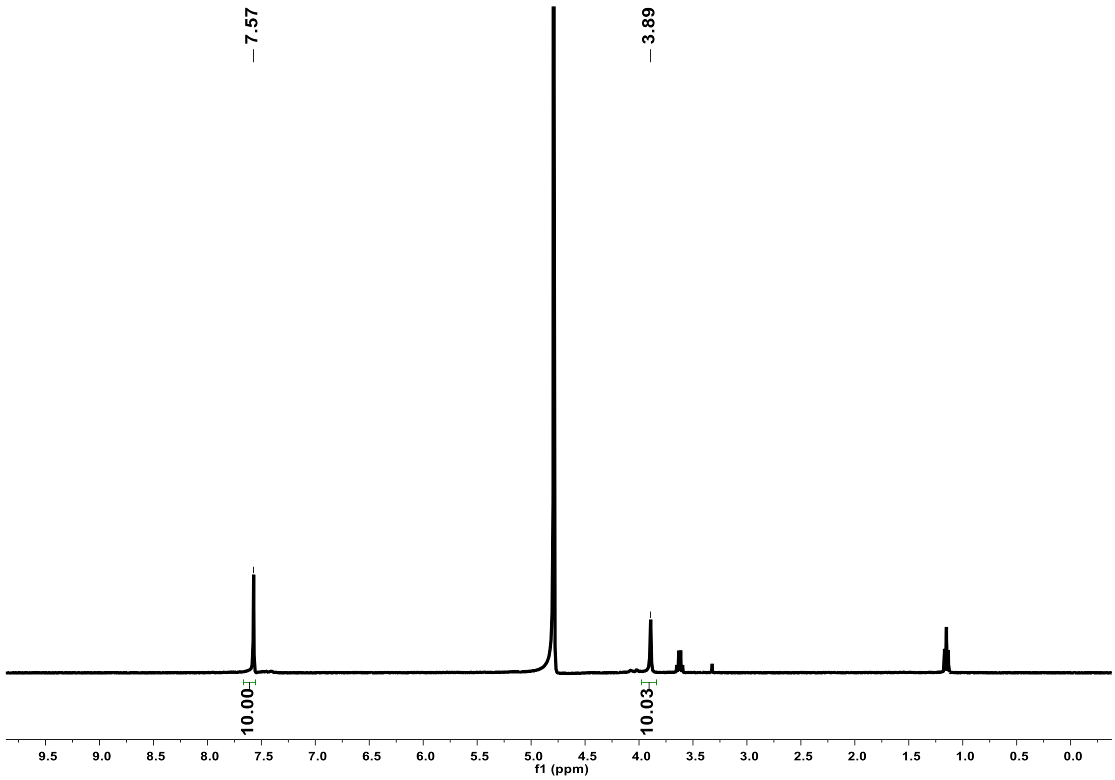

Figure S3. ^{1}H-NMR spectrum of SC5A in D_2O (400 MHz, 298.15K).

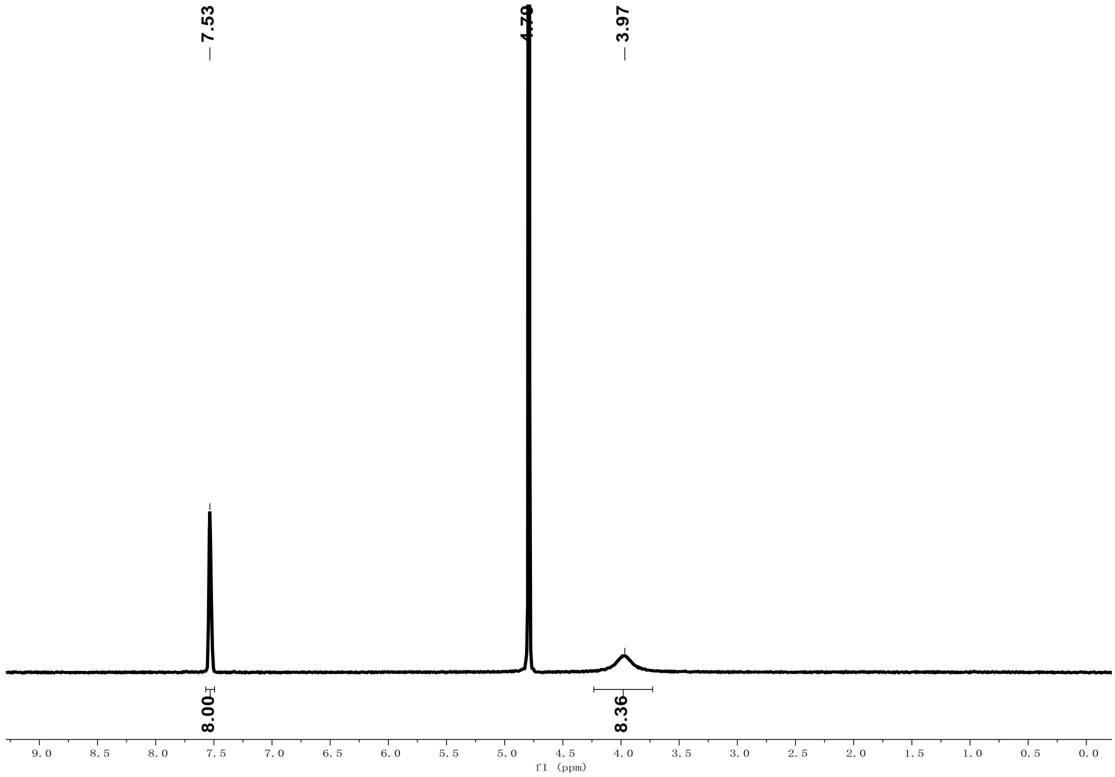

Figure S4. ^{1}H-NMR spectrum of SC4A in D_2O (400 MHz, 298.15K).

2. ITC spectra

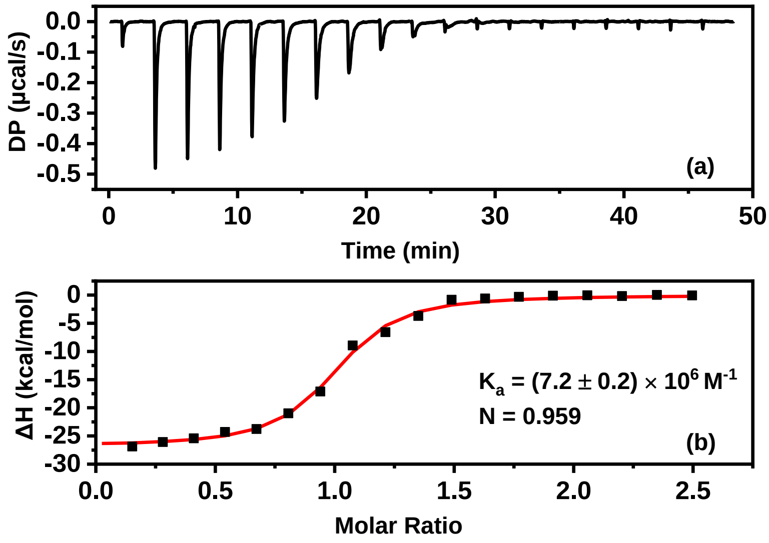

Figure S5. Calorimetric titration of GC5A (7.5 μM) with SC5A (100 μM) in HEPES buffer (10 mM, pH = 6) at 298 K. (a) Data of heat evolution with injection of SC5A. (b) Resulting binding curve (markers) and best fit (line) to a 1:1 model.

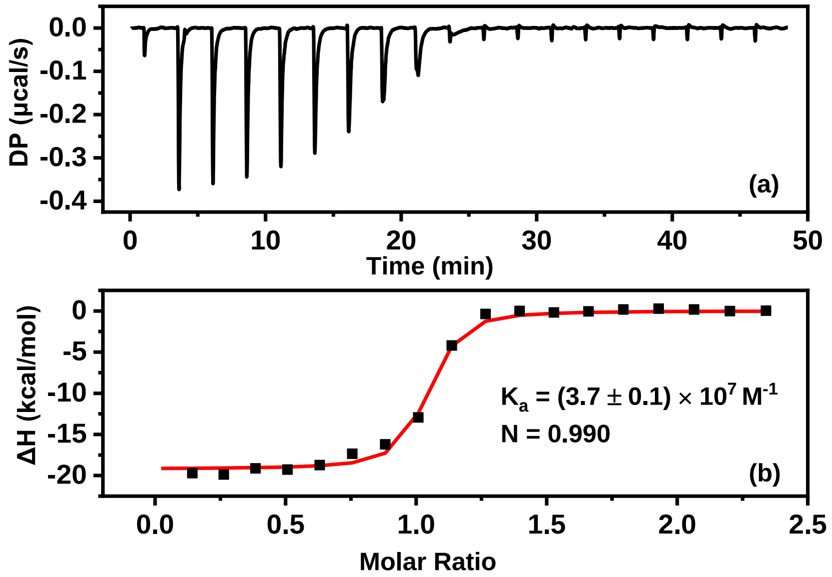

Figure S6. Calorimetric titration of GC4A (8 μM) with SC5A (100 μM) in HEPES buffer (10 mM, pH = 6.0) at 298 K. (**a**) Data of heat evolution with injection of SC5A. (**b**) Resulting binding curve (markers) and best fit (line) to a 1:1 model.

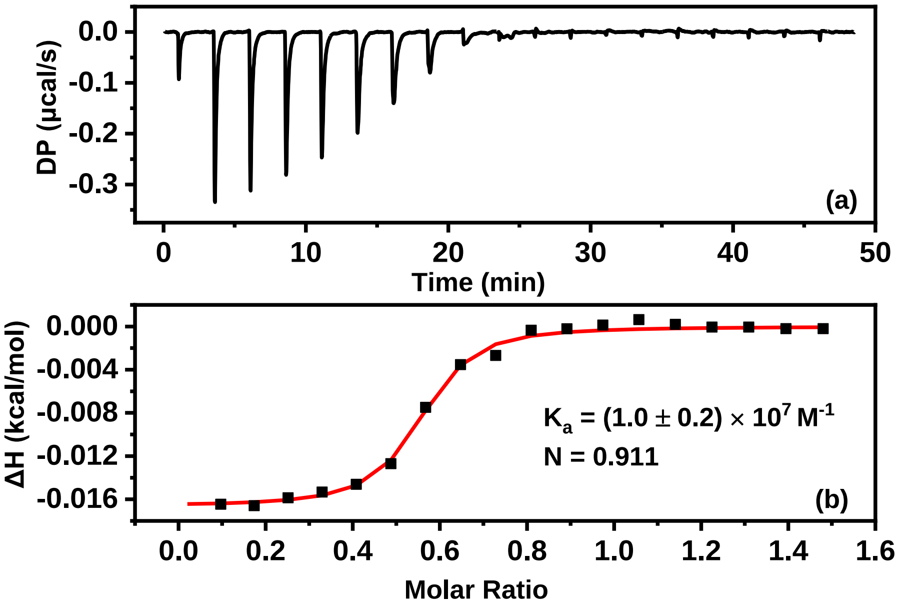

Figure S7. Calorimetric titration of GC5A (8 μM) with SC4A (100 μM) in HEPES buffer (10 mM, pH = 6.0) at 298 K. (**a**) Data of heat evolution with injection of SC4A. (**b**) Resulting binding curve (markers) and best fit (line) to a 1:1 model.

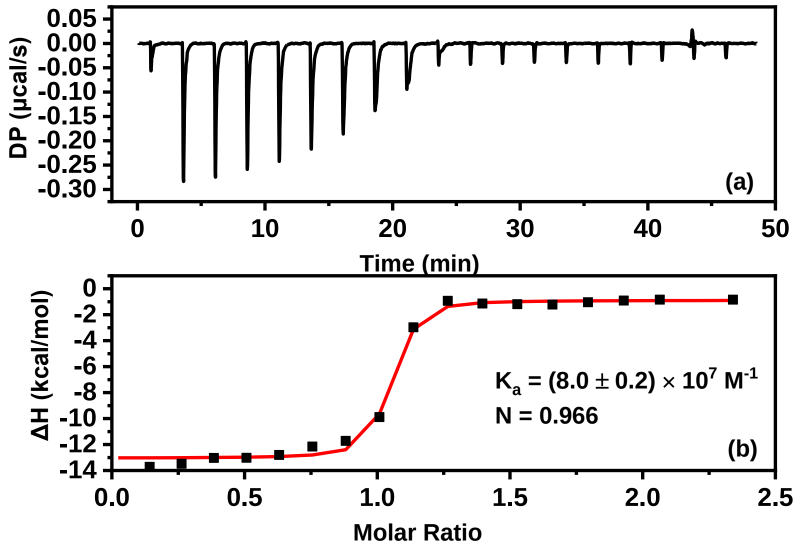

Figure S8. Calorimetric titration of GC4A (8 μM) with SC4A (100 μM) in HEPES buffer (10 mM, pH = 6.0) at 298 K. (**a**) Data of heat evolution with injection of SC4A. (**b**) Resulting binding curve (markers) and best fit (line) to a 1:1 model.

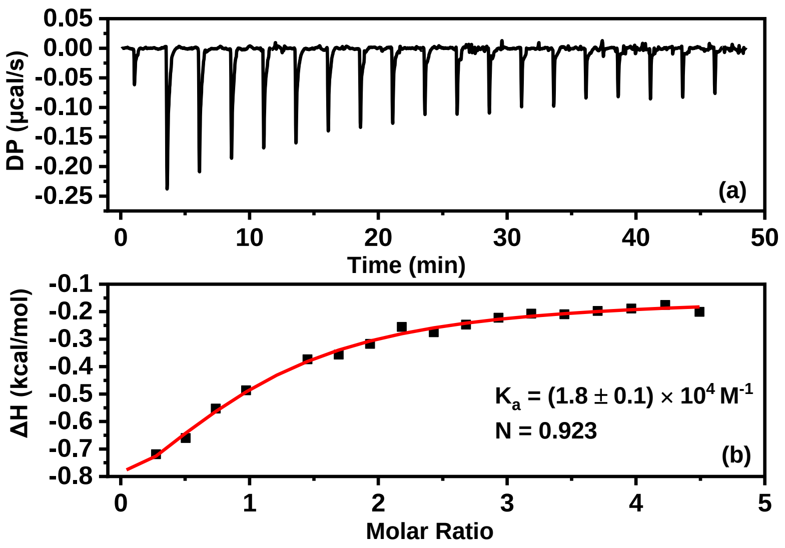

Figure S9. Calorimetric titration of SC4A (7.5 µM) with 4Asp (180 µM) in HEPES buffer (10 mM, pH = 6.0) at 298 K. (**a**) Data of heat evolution with injection of 4Asp. (**b**) Resulting binding curve (markers) and best fit (line) to a 1:1 model.

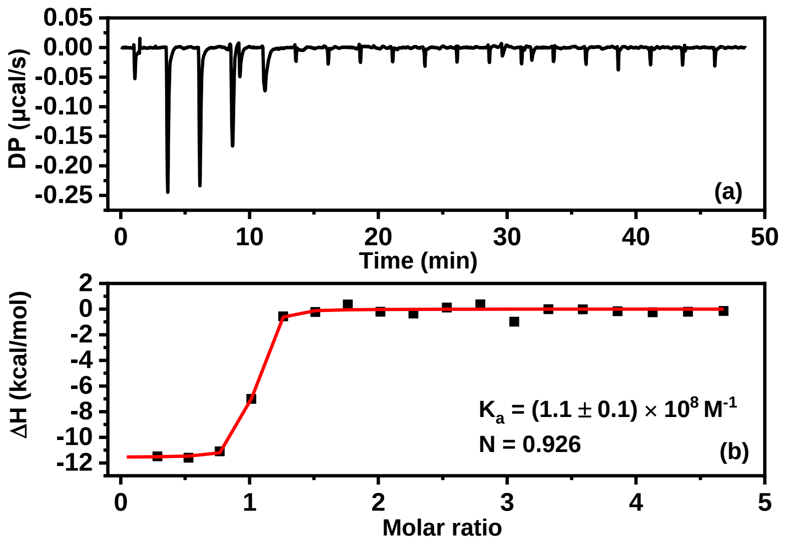

Figure S10. Calorimetric titration of GC4A (4 µM) with 4Asp@SC4A (100 µM) in HEPES buffer (10 mM, pH = 6.0) at 298 K. (**a**) Data of heat evolution with injection of 4Asp@SC4A. (**b**) Resulting binding curve (markers) and best fit (line) to a 1:1 model.

3. Fluorescence quantum yield and fluorescence spectra

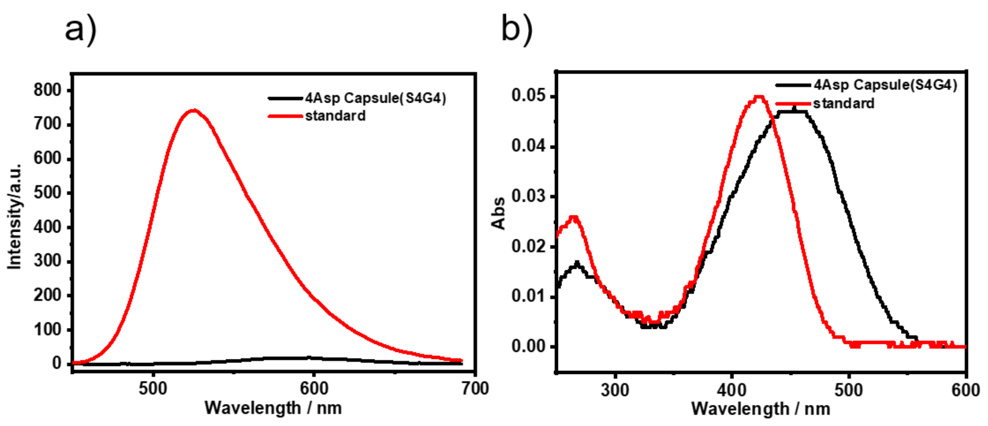

Figure S11. (**a**) Fluorescence spectra (λ_{ex} = 434 nm) of 4Asp (1.5 μM) in the presence of S4G4 (5 μM) in HEPES buffer (10 mM, pH = 6.0) and the fluorescence spectra (λ_{ex} = 434 nm) of Coumarin 153 (standard) in ethanol. (**b**) Absorption spectra of 4Asp (1.5 μM) in the presence of S4G4 (5 μM) in HEPES buffer (10 mM, pH = 6.0) and the absorption spectra of Coumarin 153 (standard) in ethanol.

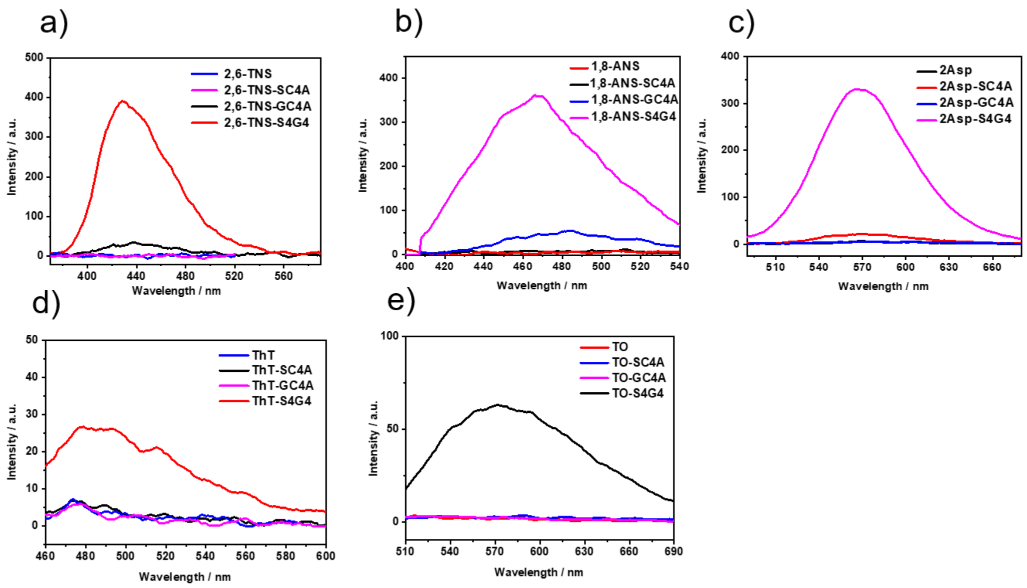

Figure S12. Fluorescence spectra of 2 μM (**a**) 2,6-TNS (λ_{ex} = 327 nm), (**b**) 1,8-ANS (λ_{ex} = 327 nm), (**c**) 2Asp (λ_{ex} = 463 nm), (**d**) ThT (λ_{ex} = 410 nm) and (**e**) TO (λ_{ex} = 488 nm) in the absence and presence of 10 μM SC4A/GC4A/S4G4, respectively. All experiments were performed in HEPES buffer (10 mM, pH = 6.0) at 298 K.

References

1. Zheng, Z.; Geng, W.-C.; Gao, J.; Wang, Y.-Y.; Sun, H.; Guo, D.-S. *Chem. Sci*. **2018**, *9*, 2087.
2. Stewart, D. R.; Gutsche, C. D. *Ore. Prep. Proc. Int.* **1993**, *25*, 137.
3. Gutsche, C.D.; Iqbal, M.; Stewart, D. *J. Org. Chem*. **1986**, *51*, 742–745
